# Association between smoking and intracranial artery dissection in patients aged less than 50 years: A propensity score-matched analysis

**DOI:** 10.18332/tid/162380

**Published:** 2023-05-18

**Authors:** Jiangli Han, Jigang Chen, Xin Tong, Mingyang Han, Fei Peng, Hao Niu, Lang Liu, Fei Liu, Aihua Liu

**Affiliations:** 1 Department of Neurosurgery, Affiliated Haikou Hospital, Xiangya School of Medicine, Central South University, Haikou, China; 2 Beijing Neurosurgical Institute, Beijing Tiantan Hospital, Capital Medical University, Beijing, China; 3 Department of Neurosurgery, the Third Xiangya Hospital, Central South University, Changsha, China; 4 Department of Neurosurgery, the Fifth Affiliated Hospital of Sun Yatsen University, Zhuhai, China

**Keywords:** angiography, atherosclerosis, dissection, risk factors, stroke

## Abstract

**INTRODUCTION:**

Smoking is a common risk factor for stroke in the young population. Intracranial artery dissection (ICAD) is a major cause of stroke in this population. However, the association between smoking and ICAD in young patients is not well characterized. We aimed to evaluate the association between smoking and ICAD in young individuals using propensity score-matched analysis.

**METHODS:**

We conducted a retrospective study of consecutive patients aged <50 years with ICAD who were admitted to Beijing Tiantan Hospital between January 2016 and December 2020. Patients with other non-atherosclerotic/non-aneurysmal cerebrovascular diseases were selected as controls. Propensity score matching was based on age and sex. Smoking and other vascular risk factors were compared between the two groups.

**RESULTS:**

The ICAD and control group included 120 and 197 patients, respectively. Propensity score matching resulted in 70 matched pairs. Smoking was the only significant factor association with ICAD in the matched cohort (p=0.031).

**CONCLUSIONS:**

In this propensity score-matched analysis, smoking showed a positive association with ICAD in young patients with common cerebrovascular diseases that were neither atherosclerotic nor aneurysmal. Further studies are required to investigate the predictive role of smoking for ICAD in the young population.

## INTRODUCTION

Cervicocranial artery dissection is a non-atherosclerotic vascular disease that can cause stroke in children and in young and middle-aged adults^1-3^. Extracranial cervical artery dissection is mainly reported in non-Asian countries, and can lead to ischemic stroke. Intracranial artery dissection (ICAD) is more prevalent in Asian countries and can result in both ischemic and hemorrhagic stroke^1^. The risk factors for extracranial cervical artery dissection have been extensively investigated^4^. Hypertension and hyperhomocysteinemia are identified as potential vascular risk factors^4,5^. However, the contemporary literature does not characterize the vascular risk factors for ICAD.

Previous studies have suggested a potential association between smoking and cervicocranial artery dissection in the young population. Smoking is highly prevalent in young stroke patients^3,6,7^. Studies have also demonstrated the role of smoking as a risk factor for ischemic and hemorrhagic stroke in young patients^8-10^. In three large-scale studies of young patients with ischemic stroke in which cervicocranial artery dissection was a significant contributor, smoking was identified as the first or second most frequent risk factor^3,11,12^. In a hospital-based cohort study, patients with vertebral artery dissection were more likely to be smokers than controls^13^. We have found that smoking is prevalent among young ICAD patients in clinical practice. ICAD and atherosclerosis are the most common causes of large-artery cerebrovascular disease in young patients^3^. Smoking is a well-known major risk factor for atherosclerotic diseases^14^. However, whether smoking is a risk factor for ICAD in the young population is not clear.

Therefore, this study evaluated the association between smoking and ICAD in young patients using propensity score-matched analysis. Given the association between smoking and atherosclerosis and the similarities between ICAD and non-dissecting intracranial aneurysm^1,15,16^, the control group enrolled in this study comprised patients with common cerebrovascular diseases neither atherosclerotic nor aneurysmal, to better control for potential confounding factors.

## METHODS

### Patients

This retrospective study enrolled consecutive patients aged <50 years with first-ever ICAD who were admitted to our hospital between January 2016 and December 2020. All diagnoses were based on clinical manifestations and radiologic findings from three-dimensional digital subtraction angiography alone or three-dimensional digital subtraction angiography combined with CT angiography, magnetic resonance angiography, and/or high-resolution magnetic resonance. An ICAD was diagnosed when the double lumen, intimal flap, or intramural hematoma was confirmed. The exclusion criteria were: 1) age ≥50 years or <18 years; 2) intracranial artery dilatation or stenosis with no signs of double-lumen, intimal flap, or intramural hematoma; 3) traumatic ICAD; 4) previously diagnosed ICAD; 5) concomitant extracranial cervical artery dissection; 6) concomitant cervicocranial atherosclerosis or non-dissecting aneurysms; 7) coronary artery disease; 8) connective tissue disease or vasculitis; and 9) lack of clinical, radiographic, or laboratory data.

### Control subjects

By reviewing the consecutive cervicocranial CT angiography data from the medical image workstation of our hospital between June 2020 and December 2020, we identified patients with cerebral arteriovenous malformations, moyamoya disease, and intracranial arteriovenous fistula. Patients for whom complete medical records were available were selected as controls. The exclusion criteria were: 1) concomitant cervicocranial artery dissection; 2) concomitant cervicocranial atherosclerosis or nondissecting aneurysms; 3) coronary artery disease; 4) connective tissue disease or vasculitis; and 5) lack of clinical or laboratory data.

### Confirmation of imaging diagnosis and data collection

For radiological inclusion and exclusion, two experienced researchers who were blinded to the clinical information independently re-interpreted the images of all potential ICAD patients and controls and arrived at consensus diagnoses. Collected data included age, sex, body mass index (BMI), smoking (use of traditional tobacco cigarettes within 6 months), hypertension, diabetes mellitus, and serum lipids (after ≥6-hour fasting). Serum lipids included triglycerides (TG), total cholesterol (TC), high-density lipoprotein cholesterol (HDL-C), low-density lipoprotein cholesterol (LDL-C), apolipoprotein AI, and apolipoprotein B.

### Statistical analysis

Continuous variables are presented as mean ± standard deviation (SD). Categorical variables are presented as frequency and percentage. The Shapiro-Wilk test was used to evaluate the normality of the distribution of continuous variables. Mann-Whitney U test was performed to compare continuous variables. Pearson’s chi-squared test was performed to compare categorical variables. Propensity score matching at 1:1 with a tolerance of 0.02 was based on age and sex. Wilcoxon rank-sum test and paired Pearson’s chi-squared test were performed to compare matched continuous and categorical variables, respectively. All analyses were performed using IBM SPSS Statistics for Windows, version 26.0 (IBM Corporation, Armonk, New York, USA). A two-tailed p<0.05 was considered indicative of statistical significance.

## RESULTS

### Comparison of patients and controls (non-matched cohort)

A total of 120 ICAD patients and 197 controls were enrolled. In the patient group, there were 88 (73.3%) males and 32 (26.7%) females, with a mean age of 40.5 ± 8.4 years (range: 18–50). In the control group, there were 100 (50.8%) males and 97 (49.2%) females, with a mean age of 28.1 ± 14.6 years (range: 2–71). The characteristics of ICAD patients and controls are compared in Table 1. ICAD patients were older and more likely to be male than controls. Smoking and hypertension were more prevalent in the patient group than in the control group. The patient group had higher levels of BMI, TG, and apolipoprotein B, and lower levels of HDLC compared to the control group. There were no significant between-group differences with respect to the prevalence of diabetes mellitus and levels of TC, LDL-C, or apolipoprotein AI.

**Table 1:** Comparison of patients and controls (non-matched cohort)

*Characteristics*	*Patients (N=120) n (%) or mean ± SD*	Controls* (N=197) n (%) or mean ± SD	*p*
Age (years)	40.5 ± 8.4	28.1 ± 14.6	<0.001
Sex, male	88 (73.3)	100 (50.8)	<0.001
BMI (kg/m^2^)	25.4 ± 4.0	22.6 ± 4.6	<0.001
Smoking	49 (40.8)	26 (13.2)	<0.001
Hypertension	49 (40.8)	29 (14.7)	<0.001
Diabetes mellitus	7 (5.8)	13 (6.6)	0.786
TG (mmol/L)	1.72 ± 1.38	1.25 ± 1.11	<0.001
TC (mmol/L)	4.25 ± 0.85	4.15 ± 0.88	0.208
HDL-C (mmol/L)	1.20 ± 0.30	1.32 ± 0.28	<0.001
LDL-C (mmol/L)	2.57 ± 0.79	2.46 ± 0.77	0.113
Apolipoprotein AI (g/L)	1.29 ± 0.25	1.30 ± 0.25	0.553
Apolipoprotein B (g/L)	0.88 ± 0.21	0.83 ± 0.20	0.006

* There are 71 controls with cerebral arteriovenous malformations, 96 controls with moyamoya disease, and 30 controls with intracranial arteriovenous fistula. BMI: body mass index. HDL-C: high-density lipoprotein cholesterol. LDL-C: low-density lipoprotein cholesterol. TC: total cholesterol. TG: triglycerides.

### Comparison of patients and controls (propensity score-matched cohort)

Propensity score matching based on age and sex resulted in 70 matched pairs. Matched comparisons are shown in Table 2. The proportion of smokers in the patient group was significantly higher than that in the control group [32.9% (23/70) vs 15.7% (11/70), p=0.031]. There were no significant differences with respect to BMI, hypertension, diabetes mellitus, TG, TC, HDL-C, LDL-C, apolipoprotein AI, and apolipoprotein B.

**Table 2:** Comparisons of patients and controls (propensity score-matched cohort)

*Characteristics*	*Patients (N=70) n (%) or mean ± SD*	*Controls (N=70) n (%) or mean ± SD*	*p*
Age (years)	37.0 ± 9.1	36.9 ± 10.2	0.899
Sex, male	38 (54.3)	39 (55.7)	>0.999
BMI (kg/m^2^)	24.4 ± 4.0	24.6 ± 3.8	0.854
Smoking	23 (32.9)	11 (15.7)	0.031
Hypertension	20 (28.6)	20 (28.6)	>0.999
Diabetes mellitus	2 (2.9)	6 (8.6)	0.289
TG (mmol/L)	1.39 ± 0.85	1.49 ± 1.49	0.986
TC (mmol/L)	4.18 ± 0.80	4.28 ± 0.88	0.542
HDL-C (mmol/L)	1.27 ± 0.33	1.31 ± 0.31	0.383
LDL-C (mmol/L)	2.53 ± 0.83	2.55 ± 0.79	0.881
Apolipoprotein AI (g/L)	1.30 ± 0.28	1.33 ± 0.27	0.661
Apolipoprotein B (g/L)	0.85 ± 0.21	0.87 ± 0.20	0.522

BMI: body mass index. HDL-C: high-density lipoprotein cholesterol. LDL-C: low-density lipoprotein cholesterol. TC: total cholesterol. TG: triglycerides.

## DISCUSSION

In this study, smoking was more prevalent in ICAD patients aged <50 years compared to those in age- and sex-matched controls with non-atherosclerotic and non-aneurysmal cerebrovascular diseases (including cerebral arteriovenous malformations, moyamoya disease, and intracranial arteriovenous fistula). Other characteristics including BMI, hypertension, diabetes mellitus, TG, TC, HDL-C, LDL-C, apolipoprotein AI, and apolipoprotein B were comparable between the patient group and the control group.

### Previous etiological investigations in the context of ICAD

As a non-atherosclerotic vascular disease, ICAD is a significant and underestimated cause of stroke in the young population^1,17^. However, the etiopathogenesis and risk factors for ICAD are not well characterized. The potential contribution of some genetic and environmental factors in the cause of ICAD was suggested in studies about cervicocranial artery dissection^1,18^. Previous studies have reported the relation of ICAD with connective tissue diseases, including fibromuscular dysplasia, Marfan syndrome, and Loeys-Dietz syndrome^19-22^. There is a paucity of studies investigating the vascular risk factors for ICAD. Moreover, no studies have compared vascular risk factors between ICAD patients and healthy controls^1^. Previous studies showed no significant differences in vascular risk factors between ICAD and cervical artery dissection^23,24^. Although some vascular risk factors for cervical artery dissection have been revealed^4,5,25^, the risk factors for ICAD have not been well investigated.

### Considerations of the study

To the best of our knowledge, this is the first study to compare vascular risk factors between ICAD patients and controls with cerebrovascular diseases that are neither atherosclerotic nor aneurysmal. Since only a few vascular risk factors have been identified for cervical artery dissection^26^, and previous studies have not shown any significant differences in vascular risk factors between ICAD and cervical artery dissection, we hypothesized that there were few vascular risk factors for ICAD, though smoking seemed promising.

Cervicocranial atherosclerosis is prevalent in a young population that may have significant vascular risk factors^3^. To reduce the potential confounding effects of common risk factors for atherosclerotic diseases and highlight the effect of smoking in a study with a relatively small sample size, we selected individuals with non-atherosclerotic cerebrovascular diseases as controls. In addition, we excluded ICAD patients or controls with cervicocranial atherosclerosis or coronary artery disease. Similarly, we excluded individuals with non-dissecting cervicocranial aneurysms to reduce the potential confounding effects of aneurysm-associated vascular and genetic risk factors. Cerebral arteriovenous malformations, moyamoya disease, and intracranial arteriovenous fistula are neither atherosclerotic nor aneurysmal diseases. As they are prevalent in the young population and lack common vascular risk factors, individuals with these diseases are suitable as controls for this study.

### Smoking and ICAD

ICAD is thought to be a multifactorial arteriopathy involving both environmental and genetic factors. Gene-related structural defects of the arterial wall may be predisposing factors for ICAD^1,13,18,27^. As a major preventable risk factor for various vascular diseases, smoking may play a role in ICAD. Previous studies involving ICAD patients have suggested a potential association between smoking and ICAD. A study of 301 patients with cervicocranial artery dissection provided evidence of the potential association between smoking and cervicocranial artery dissection; 95 of 301 patients in this study had intracranial dissection or intracranially extending dissection^28^. In a case-control study involving 112 patients with vertebral artery dissection and 224 controls primarily with carotid-cavernous fistula, cerebral venous sinus thrombosis, or cerebral arteriovenous malformation, smoking showed an independent association with vertebral artery dissection^13^. Daou et al.^29^ reported an association of smoking with the progression of carotid and vertebral artery dissection. Smoking is a leading risk factor for atherosclerosis. However, smoking can be even more prevalent in patients with vertebrobasilar artery dissection but without cervical or cerebral artery atherosclerosis than in patients with both vertebrobasilar artery dissection and cervical or cerebral artery atherosclerosis^30^.

The mechanisms by which smoking may increase the propensity for ICAD are unknown. ICAD occurs in the context of a weakened arterial wall related to a predisposition to connective tissue disease. Dissecting aneurysm is very common in ICAD1, where the pathological changes are similar to those in non-dissecting aneurysm^15^. As smoking increases the risk for non-dissecting intracranial aneurysms in patients with connective tissue disease^31,32^, it probably increases the risk of ICAD in the young population with a predisposition for connective tissue disease with a similar mechanism. Smoking-related endothelial dysfunction, inflammation, thrombotic tendency, hemodynamic changes, and alterations of gene expression may contribute to ICAD as they do to the formation of non-dissecting aneurysms^33,34^.

Combining our findings with previously reported results, we speculate that the interaction between smoking and genetic predisposition for connective tissue disease may play an important role in the pathogenesis of ICAD.

### Strengths and limitations

Some limitations of our study should be acknowledged. First, the retrospective single-center study design may have introduced potential bias. Second, passive smoking is a common vascular risk factor in the young population, similar to active smoking^35^. However, we did not evaluate the association between passive smoking and ICAD, owing to the lack of adequate data in the medical records. Third, age and sex are potential critical factors for ICAD^1,28^. Nonetheless, their impact was not investigated as this study entailed a propensity score-matched analysis based on age and sex. Fourth, as no data regarded the amount of smoking, the dose-dependent relationship was not assessed. Fifth, we did not evaluate hyperhomocysteinemia as a potential confounding factor for ICAD. Sixth, selection bias may have been introduced due to the loss of ICAD patients after matching. Seventh, the generalizability of our findings may be restricted due to the limitations of control selection.

## CONCLUSIONS

Smoking was found to be associated with ICAD in patients aged <50 years with common cerebrovascular diseases that were neither atherosclerotic nor aneurysmal. Further studies are required to investigate the predictive role of smoking for ICAD in the young population.

## Data Availability

The data supporting this research are available from the authors on reasonable request.
